# Adherence to Antibiotic Prescription of Dental Patients: The Other Side of the Antimicrobial Resistance

**DOI:** 10.3390/healthcare10091636

**Published:** 2022-08-27

**Authors:** Francesco D’Ambrosio, Federica Di Spirito, Francesco De Caro, Antonio Lanza, Daniela Passarella, Ludovico Sbordone

**Affiliations:** 1 Department of Medicine, Surgery and Dentistry “Schola Medica Salernitana”, University of Salerno, 84081 Salerno, Italy; 2 Department of Medicine and Health Sciences “V. Tiberio”, University of Molise, 86100 Campobasso, Italy

**Keywords:** antibiotics, antibiotic stewardship, bacterial resistance, Morisky scale, patient adherence

## Abstract

Since non-adherence to antibiotic therapy can cause several problems, including antimicrobial resistance (AMR) and treatment failures, the present study evaluated adherence to oral antibiotic therapy and AMR awareness among consecutively enrolled dental patients. Data concerning age, gender, socioeconomic status, education level, cohabitation, and general health were retrieved from medical records. AMR awareness was investigated through direct questions and adherence to antibiotic treatment was assessed through a modified Italian version of the Morisky medical scale-8 items. Participants’ characteristics were analyzed in relation to treatment adherence and AMR, using a Χ^2^ independence test (significance level of α <0.1). Dental patients generally showed a low (51.82%) adherence to oral antibiotic therapy, and medium and high adherence was reported only by 29.37% and 18.81% of participants. Treatment adherence was similar in relation to participants’ gender and age but significantly lower in subjects with only secondary school graduation and higher in participants with higher education levels. Non-cohabitants were significantly more adherent than cohabitants. AMR awareness was declared by 42.15% of males and 38.70% of females: 56.52% of dental patients aware of AMR were 18–38 years old, 35.20% were 39–59 years old, and 26.95% were aged between 60 and 80. Further studies are needed to develop adequate strategies, expanding dental patients’ knowledge of AMR, thus optimizing the benefits and reducing the risks of antibiotic administration in dental patients.

## 1. Introduction

The discovery and the use of antibiotics was one of the most important revolutions of the twentieth century to control and reduce infectious diseases [1].

However, excessive use and misuse of antibiotics play an important role in increasing bacterial resistance [2], leading, in turn, to an increased risk of complications and mortality and, consequently, to a more complex resolution of infectious diseases and potential treatment failure. Moreover, the overuse of antimicrobials increases healthcare costs [3,4], thereby causing a global public health problem that crosses all borders and is present worldwide [5,6].

Antimicrobial resistance (AMR) is caused by several factors, including inappropriate antibiotic prescription with overuse or misuse, and poor patient adherence to antimicrobial therapy, non-compliant with treatment recommendations [7], registering an increase in the improper use of antibiotics across Europe in recent years. Out of all antibiotics administrations, 7–10% were in an outpatient environment, with dentistry accounting for a comparatively higher amount of these prescriptions [7], as confirmed by numerous findings highlighting that dentists often do not observe antibiotics prescription guidelines, especially for prophylactic purposes in dentoalveolar surgery [8,9,10].

In addition, according to a recent global survey, non-adherence to antibiotic therapy, which determines an inappropriate use and may increase the risk of therapeutic failure, reinfection, and AMR, was estimated at around 22.3%, with a variation between 9% and 44% among countries [11]. Consequently, several measures were undertaken to improve patients’ adherence to short-term antimicrobial treatments, comprising accurate antibiotic prescriptions and written information on the importance of taking all medications [12]. To assess patients’ adherence to antibiotics, different direct and indirect methods, characterized by specific advantages and disadvantages, were developed [13,14], among which the Morisky Medication Adherence Scale (MMAS-4) is one of the most common indirect methods employed [15,16,17,18]. Based on the MMAS-4, the Morisky Medication Adherence Scale-8 (MMAS-8), which is an 8-item self-reported scale [19], was developed, and its validated Italian version [20], amended accordingly, was employed in the present study.

Considering the contributing role of patients’ adherence to antibiotic treatments in AMR [11,12] and the insufficient evidence concerning dental prescription, the study primarily aimed to assess the adherence to oral antibiotic therapy through a modified Italian version of the Morisky Medical Adherence Scale-8 items, also in relation to participants’ characteristics, and secondarily to evaluate the awareness of antimicrobial resistance among dental patients.

## 2. Materials and Methods

### 2.1. Study Design and Eligibility Criteria

Outpatients (not hospitalized) attending the Complex Operating Unit of Odontostomatology, Head and Neck Clinical Department, between January and July 2021, who provided written informed consent and met the eligibility criteria described below, were consecutively enrolled in the study. The sample size was obtained from a previous study evaluating patients’ adherence to oral antibiotic prescription in outpatients [21,22,23].

Inclusion criteria were mentally competent subjects aged between 18 and 80 with no gender restriction, who received an antibiotics prescription compliant with the guidelines. Exclusion criteria were mentally incompetent subjects or subjects with cognitive impairments and chronic diseases causing worsening of the cognitive sphere, or those younger than 18 years of age.

### 2.2. Data Collection and Adherence to Antibiotic Treatment Assessment

Data concerning age, gender, socioeconomic status, education level, cohabitation, and general health were retrieved from medical and dental records.

In this study, only orally administered antibiotics were considered, being more frequently prescribed in dentistry compared to the other administration routes and characterized by higher compliance to treatment [24].

Upon completion of the oral antibiotics treatment prescribed, awareness of antibiotic resistance was investigated through direct questions asked to all participants, and adherence to the antibiotic prescription was assessed through a closed questionnaire capable of determining adherence to antibiotic therapy delivered to all enrolled subjects. The currently employed questionnaire was a validated Italian version [20] of the Morisky Medical Adherence Scale-8 items, adapted for a short-term oral antibiotic treatment; accordingly, questions 2 and 5 were properly modified. Question 2 originally asked, “People sometimes miss taking their medications for reasons other than forgetting. Over the past 2 weeks, were there any days when you did not take your medications?”It was modified as:“People sometimes miss taking their medications, but not because they forget it. Were there any days when you didn’t take antibiotics?” Question 5 originally asked,“Did you take all your medications yesterday?”It was modified to:“Did you take antibiotics on the last day of therapy?” (Table 1).

Questionnaire items 1–7 required a yes or no answer, while item 8 provided a Likert scale answer (never, rarely, sometimes, often, always). As previously described [22], the questionnaire score was computed as follows: one point was assigned to no answers, and no points were assigned to yes answers, except for item 5, where the scores were inverted (no points were assigned to no answers, one point was assigned to yes answers). For item 8, the answers never and rarely were assigned a value of 1, while the answers sometimes, often, and always were assigned a value of 0. The total questionnaire score ranged from 0 to 8, where a score of 8 indicated complete adherence, scores of 6–7 showed medium adherence, and scores < 6 indicated a low adherence to antibiotic therapy [22].

### 2.3. Statistical Analysis

A descriptive statistical analysis of participants’ socio-demographic characteristics, antibiotic treatment adherence, and antimicrobial resistance awareness was conducted, to analyze and summarize collected data using Microsoft Excel software 2019 (Microsoft Corporation, Redmond, WA, USA). Socio-demographic characteristics were evaluated in relation to the level of antibiotic treatment adherence through a Χ^2^ independence test with a significance level of α < 0.01.

## 3. Results

### 3.1. Study Population

From January to July 2021, a total of 730 patients were examined, and 598 patients satisfied the inclusion and exclusion criteria.

Out of the 598 potentially eligible subjects, 45 did not give their consent to participate in the study, and 32 arbitrarily refused the antibiotic therapy prescribed; consequently, 521 patients were finally recruited for the study.

Socio-demographic characteristics of the enrolled subjects are summarized in Table 2.

### 3.2. Education Level and Cohabiting

In total,255 subjects were cohabitants, and among them, 25 (9.80%) had an elementary school license, 82 (32.16%) had secondary school graduation, 100 (39.22%) had high school graduation, and 48 (18.82%) had a university degree.

Among the 266 non-cohabitants, 8 (3.01%) had an elementary school license, 29 (10.90%) had secondary school graduation, 140 (52.63%) had high school graduation, and 89 (33.46%) possessed a university degree.

### 3.3. Antibiotic Therapy Adherence

**Figure 1:**
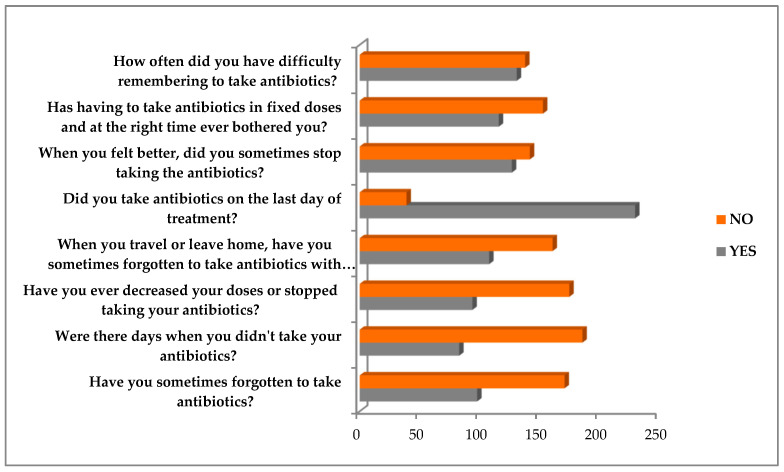
Distribution of the Morisky scale score based on the questions and answers.

Based on the Morisky Medical Adherence Scale-8 items questionnaire, the score distribution based on the reported answers is shown in Figure 1.

**Figure 2:**
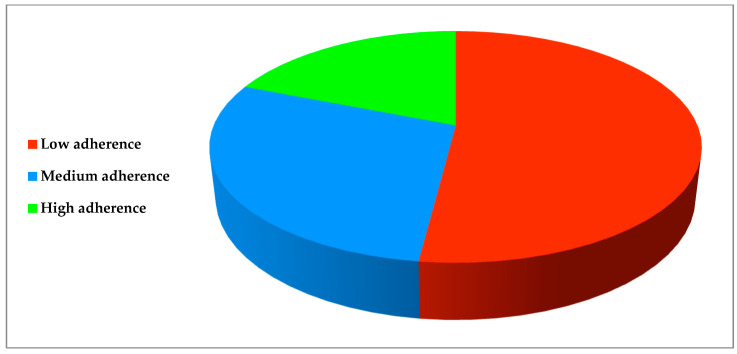
Participants’ adherence to antibiotic treatment.

The overall level of adherence resulted as low in 270 (51.82%) subjects, medium in 153 (29.37%), and high in 98 (18.81%), respectively (Figure 2).

The Χ^2^ independence test (significance level of α < 0.01) revealed no statistically significant (α < 0.01) differences in the level of antibiotic treatment adherence in relation to participants’ gender and age. Conversely, a significantly lower adherence to therapy was found in subjects (n = 69) with secondary school graduation and a significantly higher adherence in those(n = 86) with high school graduation or a university degree. Non-cohabitants were significantly more adherent to antibiotic therapy when compared to cohabitants. The distribution of adherence levels based on gender, age, education level, and cohabiting is summarized in Table 3.

### 3.4. Antibiotic Resistance (AMR) Awareness

Awareness of antibiotic resistance was declared by 110 males (42.15%) and 101 females (38.70%), and by 104 subjects (56.52%) between 18 and 38 years old, 69 (35.20%) between 39 and 59 years of age, and 38 (26.95%) of those aged between 60 and 80.

In relation to the education level, AMR awareness was expressed by 2 subjects(6.06%) with elementary school license, 20 (18.02%) with secondary school graduation, 93 (38.75%) with high school graduation, and 96 (70.07%) university-graduated subjects. Among the 211 subjects who declared to be aware of AMR, the level of adherence to antibiotic treatment resulted low in 66 subjects (31.28%), medium in 83 (39.34%), and high in 62 (29.38%), respectively.

Analyzing the socio-demographic characteristics of enrolled subjects in relation to the awareness of antibiotic resistance, no association (α = 0.01) was found concerning participants’ gender; conversely, a statistically significant association (α = 0.01) was found in younger compared to older subjects. A significant association was also found between subjects with higher compared to lower education levels (α = 0.01) and between those showing higher adherence to antibiotic prescription (α = 0.01) and AMR awareness.

## 4. Discussion

The present study primarily aimed to assess the adherence to oral antibiotic therapy through a modified Italian version of the Morisky Medical Adherence Scale-8 items, also in relation to participants’ characteristics, and secondarily to evaluate the awareness of antimicrobial resistance among dental patients, in view of the contributing role of patients’ adherence to antibiotic treatments in AMR [11,12] and of the insufficient evidence concerning oral prescription in dentistry.

Adherence to the prescribed oral antibiotic therapy resulted as low in 270 (51.82%) dental patients, medium in 153 (29.37%), and high in 98 (18.81%). Our results are in accordance with an Italian study, revealing a low adherence to antibiotic treatment in 59.9% of the study population [25], and another study conducted in Europe, reporting that 55.8% of participants did not complete the antimicrobial therapy prescribed [26]. Similarly, Llor et al. described a non-adherence of 25.2% in a Spanish sample, progressively declining over time in an additional 28.7% [27]; in a Chinese study, adherence to antibiotic therapy was observed in only 13% of patients [28].

In the present study, the level of adherence to antibiotic therapy was negatively influenced by cohabitation, in contrast with Desta et al. results [29], and positively linked to a higher education level of the participants. The last finding may be explained by the fact that currently enrolled, non-cohabiting subjects had a higher education level (52.63% with high school graduation and 33.46% with a university degree) than cohabiting ones (39.22% with high school graduation and 18.82% with a university degree).

No relation has been presently found between the level of adherence to antibiotic therapy and gender, compliant with the results of a recent meta-analysis [30]; however, conflicting results have been obtained by Manteuffel et al. [31], reporting lower adherence in the female gender.

Our results showed no significant differences in adherence to antibiotic treatment between participants in the different age groups. In contrast, Fernandes et al. identified increasing age as a factor associated with non-adherence [32], while Rolnick et al. found a greater non-adherence in younger patients [33].

Awareness of antimicrobial resistance resulted aslow in 66 dental patients (31.28%), medium in 83 (39.34%), and high in 62 (29.38%). AMR was positively associated with higher education levels and a younger age. Indeed, it appears that older age groups have a lower AMR awareness, and that the failure of antibiotic intake is mainly caused by forgetfulness in 37.24% of cases, compared to 30.71% who do not take it intentionally [34]. More generally, 36.08% of patients stopped taking or decreased the doses of their antibiotics, and 48.37% stopped taking their antibiotics in advance as their health conditions improved. Less adherence to antibiotic therapy was found associated with a lower level of education and knowledge of the issues related to the improper use of antibiotics. The results from this study agree with a recent study by Chan et al., concluding that the lack of knowledge of antibiotics, including the problem related to antimicrobial resistance, affects adherence to antibiotic therapy. Therefore, patients, unaware of the possible damage to both their health and the public health, may pay less attention to following antibiotic treatment or even intentionally decide not to adhere to it correctly [35]. Similarly, Raupach-Rosin et al. pointed out that higher education, especially a university degree, positively affects knowledge and AMR awareness [36]. However, such findings highlight the need to provide dental patients with more accurate indications on antibiotic prescription and information on antimicrobial resistance, aiming primarily to increase knowledge and comprehension of the benefits related to the correct use of antibiotics as well as of the risks deriving from their abuse and misuse, and secondarily, to improve dental patients’ overall behavior toward antibiotic intake. According to these considerations, the present study also noted a significant positive association between AMR and adherence to antibiotic therapy.

Moreover, by simplifying antibiotic administration, in compliance with guidelines for antimicrobial stewardship, dental patients could better tolerate the prescribed treatment regimen, especially considering that 51.63% of participants showed difficulty remembering to take their antibiotics [37,38,39,40,41]. Today, the adherence to antibiotic therapy has increased thanks to electronic devices with a reminder function, smartphone apps, and periodic adherence measurements [42,43,44]. Other simply modifiable adherence barriers, mainly described as worries about side effects and drug–drug interactions, and difficulties in swallowing tablets and integrating antibiotic intake into daily life, should also be carefully addressed to optimize patients’ adherence to antibiotic treatment [45]. Finally, the presented results, revealing a low level of adherence to antibiotic therapy in dental patients, are consistent with the findings from other studies employing a self-report as a method for measuring adherence [42].

The limited number of enrolled participants may represent the main limitation of the study, not allowing the generalizability of the results with a high level of accuracy. Therefore, consistent conclusions could not be drawn and presented findings definitely require validation by more comprehensive studies. However, to our knowledge, the present study is the first to evaluate the adherence to antibiotic therapy in dental patients. The Morisky scale questionnaire, currently employed, allowed a sensitive measurement of the adherence to antibiotic treatment, also reflecting dental patients’ behavior during antimicrobial intake [46]. Although self-reports have well-known intrinsic limitations due to memory bias, overestimating adherence, and stimulation of socially acceptable responses [46], assessing adherence to antibiotic therapy through questionnaires seems to have a medium–high agreement with measurements obtained through other methods [20,47,48,49].Its use, combined with additional objective assessment methods, such as electronic adherence monitoring devices, pill counts, pharmacy refill rate, or direct drug and biomarker measurement, may be indicated to improve measurements [20,44].

## 5. Conclusions

Presently enrolled dental patients generally showed a low (51.82%) adherence to oral antibiotic therapy, significantly higher in participants with higher education levels and in non-cohabitants. Given that adherence to antibiotic treatment is necessary to make the therapy effective and, at the same time, to reduce the impact of antimicrobial resistance on public health, interventions to expand dental patients’ knowledge of antibiotic therapy and, particularly, of antimicrobial resistance, may enhance their adherence to treatment.

Antimicrobial resistance awareness was higher in younger (56.52%)dental patients. Such findings suggest that focused interventions counteracting patients’ non-adherence, especially eliminating modifiable adherence barriers and improving AMR knowledge, should be directed to the elderly.

Further studies are needed, due to the lack of investigations on adherence to antibiotic treatment in dentistry, to assess it and to evaluate possible influencing factors to develop adequate strategies to optimize the benefits and reduce the risks of antibiotic therapies in dental patients.

## Figures and Tables

**Table 1:** Administered questionnaire assessing adherence to antibiotic therapy: modified version of the Morisky Medical Adherence Scale-8 items (MMAS-8) [19].

1. Have you sometimes forgotten to take antibiotics?	No	Yes
2. Some people sometimes skip taking their medications, but not because they forget. Were there days when you didn’t take your antibiotics?	No	Yes
3. Have you ever decreased your doses or stopped taking your antibiotics?	No	Yes
4. When you travel or leave home, have you sometimes forgotten to take antibiotics with you?	No	Yes
5. Did you take antibiotics on the last day of treatment?	No	Yes
6. When you felt better, did you sometimes stop taking the antibiotics?	No	Yes
7. For some people taking medication is a real hassle. Has having to take antibiotics in fixed doses and at the right time ever bothered you?	No	Yes
8. How often did you have difficulty remembering to take antibiotics?	Never/Rarely Occasionally	Often Always Sometimes

**Table 2:** Socio-demographic characteristics of the studied population.

Participants	*n*	%
Gender		
▪ Male	261	50.10%
▪ Female	260	49.90%
Age		
▪ 18–38	184	35.32%
▪ 39–59	196	37.62%
▪ 60–80	141	27.06%
Education level		
▪ Elementary license	33	6.33%
▪ Secondary school graduation	111	21.31%
▪ High school graduation	240	46.07%
▪ University degree	137	26.30%
Cohabiting		
▪ Cohabiting	255	48.94%
▪ Non-cohabiting	266	51.06%

**Table 3:** Distribution of adherence levels by gender, age, education level, and cohabiting.

Participants	Low Adherence	Medium Adherence	High Adherence
Gender			
▪ Male	127	85	49
▪ Female	143	68	49
Age			
▪ 18–38	90	49	45
▪ 39–59	100	62	34
▪ 60–80	80	42	19
Education level			
▪ Elementary license	21	7	5
▪ Secondary school graduation	69	35	7
▪ High school graduation	125	63	52
▪ University degree	55	48	34
Cohabiting			
▪ Cohabiting	145	76	34
▪ Non-cohabiting	125	77	64

## Data Availability

Not applicable.
